# Prothrombotic gene variants as risk factors of acute myocardial infarction in young women

**DOI:** 10.1186/1479-5876-10-235

**Published:** 2012-11-21

**Authors:** Rossella Tomaiuolo, Chiara Bellia, Antonietta Caruso, Rosanna Di Fiore, Sandro Quaranta, Davide Noto, Angelo B Cefalù, Pierpaolo Di Micco, Federica Zarrilli, Giuseppe Castaldo, Maurizio R Averna, Marcello Ciaccio

**Affiliations:** 1 CEINGE-Biotecnologie Avanzate, Via Gaetano Salvatore 486, Naples, 80145, Italy; 2 Dipartimento di Biochimica e Biotecnologie Mediche, Università di Napoli Federico II, Naples, Italy; 3 Facoltà di Scienze Biotecnologiche, Università di Napoli Federico II, Naples, Italy; 4 Sezione di Biochimica Clinica e Medicina Molecolare, Dipartimento di Biopatologia e Biotecnologie Mediche e Forensi, Università di Palermo, Palermo, Italy; 5 Centro per le Dislipidemie Genetiche-Dipartimento di Medicina Interna e Specialistica, Università di Palermo, Via del Vespro 129, Palermo, 90127, Italy; 6 Pronto Soccorso, Unità di Medicina Interna, Ospedale Fatebenefratelli di Napoli, Naples, Italy; 7 Dipartimento di Bioscienze e Territorio, Università del Molise, Isernia, Italy

**Keywords:** Young AMI, Gender, AMI, Gene variants, Mutations, Prothrombotic variants, Genetic predisposition

## Abstract

**Background:**

Acute myocardial infarction (AMI) in young women represent an extreme phenotype associated with a higher mortality compared with similarly aged men. Prothrombotic gene variants could play a role as risk factors for AMI at young age.

**Methods:**

We studied Factor V Leiden, FII G20210A, MTHFR C677T and beta-fibrinogen -455G>A variants by real-time PCR in 955 young AMI (362 females) and in 698 AMI (245 females) patients. The data were compared to those obtained in 909 unrelated subjects (458 females) from the general population of the same geographical area (southern Italy).

**Results:**

In young AMI females, the allelic frequency of either FV Leiden and of FII G20210A was significantly higher versus the general population (O.R.: 3.67 for FV Leiden and O.R.: 3.84 for FII G20210A; p<0.001). Among AMI patients we showed only in males that the allelic frequency of the MTHFR C677T variant was significantly higher as compared to the general population. Such difference was due to a significantly higher frequency in AMI males of the MTHFR C677T variant homozygous genotype (O.R. 3.05).

**Discussion and conclusion:**

Our data confirm that young AMI in females is a peculiar phenotype with specific risk factors as the increased plasma procoagulant activity of FV and FII. On the contrary, the homozygous state for the 677T MTHFR variant may cause increased levels of homocysteine and/or an altered folate status and thus an increased risk for AMI, particularly in males. The knowledge of such risk factors (that may be easily identified by molecular analysis) may help to improve prevention strategies for acute coronary diseases in specific risk-group subjects.

## Background

Given its high incidence, morbidity and mortality, acute myocardial infarction (AMI) is a relevant clinical and social problem making heart disease a leading killer particularly in young women [1,2]. Importantly, registries and cohort studies revealed an excess mortality risk following AMI in young women compared with similarly aged men [3]. The higher risk may be due to a higher prevalence of various traditional and emerging risk factors in this group compared with men. Data on risk factors for young AMI may help to improve strategies for its prevention. In a relevant percentage of cases, young AMI occurs independently of the presence of typical risk factors such as dyslipidemia, smoking, hypertension, diabetes. In such cases, familiarity for AMI is frequently observed suggesting that genetic predisposition plays a relevant role [4].

Prothrombotic gene variants were found in genes involved in haemostasis and its inhibition, leading to a procoagulant effect. The most common prothrombotic variants are: Factor V (FV) Leiden, which causes resistance to protein C; prothrombin (FII) G20210A variant, that causes a higher plasma level of FII; the C677T variant of methylene-tetrahydrofolate reductase (MTHFR) enzyme which impairs the homocysteine pathway thereby causing higher serum levels of homocysteine that acts as a trigger for coagulation at endothelial level; and the -455G>A variant of beta-fibrinogen which causes increased levels of fibrinogen [5]. Prothrombotic gene variants are already known to be risk factors for recurrent venous thrombosis [6], particularly in patients with other predisposing disease [7] whereas there is no consensus regarding the role of such variants as risk factors for arterial disorders, including AMI and young AMI [8-12]. The discordant results reported so far may reflect heterogeneous selection criteria and the different number of AMI patients and controls analyzed in different studies [13], and the fact that most studies pooled male and female patients thereby obscuring eventual gender differences. The different results may also depend on the variable incidence of prothrombotic variants in subjects of different ethnic background, and on the fact that some studies compared the frequency of prothrombotic gene variants obtained in AMI patients with that of control populations of other geographical areas.

The aim of the present study was to evaluate the potential role of the four prothrombotic gene variants as risk factors of either young AMI and AMI in female and male subjects in comparison to a large group of individuals from the general population of the same geographic area, whose sex and age distribution corresponded to the anagraphic distribution of the population of southern Italy.

## Materials and methods

### Subjects

We studied: i) 955 young AMI patients (362 females and 593 males), mean age: 38.7 years; and ii) 698 AMI patients (245 females and 453 males), mean age: 69.5 years. All patients were from southern Italy at least from three generations.

Acute myocardial infarction was diagnosed according to the Joint ESC/ACCF/AHA/WHF Task Force for the Redefinition of Myocardial Infarction [14]. AMI patients had their first episode at an age above 45 years, whereas young AMI patients had their first episode before the age of 45 years. All patients were negative for previous AMI and for other major ischemic diseases such as ischemic stroke or lower limb ischemia. Each patient released informed consent to their blood sample and clinical data being used for anonymous scientific studies on genetic susceptibility to AMI. A blood sample was obtained from each patient during a routine visit.

The data of prothrombotic variants obtained from AMI and young AMI patients were compared to those of a group from the general population of southern Italy constituted by 909 unrelated subjects (458 females and 451 males; mean age: 54 years) from southern Italy up to the third generation. The sex and age distribution of this group (see Table 1) matched the anagraphic distribution of subjects in southern Italy. For most of the subjects from the general population, DNA samples were already available in the bank of biological samples from the institutions involved in the study, and are currently used collectively for the study of gene variants associated with inherited diseases [15,16].

**Table 1:** Number and allelic frequency (%) for prothrombotic gene variants in males and females from the general population

**Group**	**FV Leiden**	**FII G20210A**	**MTHFR C677T**	**Beta Fibrinogen -455G>A**
Female subjects	21/870 (2.4%)	22/870 (2.5%)	395/916 (43.1%)	108/744 (14.5%)
Male subjects	21/848 (2.5%)	21/848 (2.5%)	365/902 (40.5%)	78/564 (13.8%)

This study was performed according to the ethical requirements of the institution, and the informed consent was obtained from each individual.

### Methods

The DNA for the analysis of prothrombotic gene variants was extracted from leukocytes using a commercial kit (Nucleon BACC2, Amersham Biosciences, Little Chalfont, UK). The four prothrombotic gene variants, i.e., Factor V Leiden, FII G20210A, MTHFR C677T and -455G>A in the gene encoding beta fibrinogen were analyzed using a specific commercial kit based on real-time PCR and ligth-cycler analyzer (Roche, Monza, Italy). For FIIG20210A, MTHFR C677T and -455G>A variant, the variant alleles were respectively: “A”, “T” and “A”.

### Statistics

To compare i) the distribution of allele and genotype frequencies in the different age groups and sex of control subjects from the general population and ii) the distribution of allele and genotype frequencies of the four gene variants obtained in patients and in control subjects we used the Yates’ chi square test. A p level <0.001 was considered significant.

## Results

The three populations of our study resulted in the Hardy-Weinberg equilibrium for all tested gene variants.

### General population

Table 1 reports the data of allelic frequency for the four prothrombotic variants (FV Leiden, FII G20210A, MTHFR C677T and -455G>A of beta fibrinogen) in subjects from the general population. No significant differences were obtained between males and females for any of the variables. Furthermore, we compared the distribution of the allelic frequencies for the four variables in different classes of ages (i.e., < 20 yrs, 20 to 40 yrs, 40 to 60 yrs and > 60 yrs). Again no differences were recorded (data not shown); thus all subjects from the general population were pooled together in next comparisons.

### Young AMI patients

**Figure 1:**
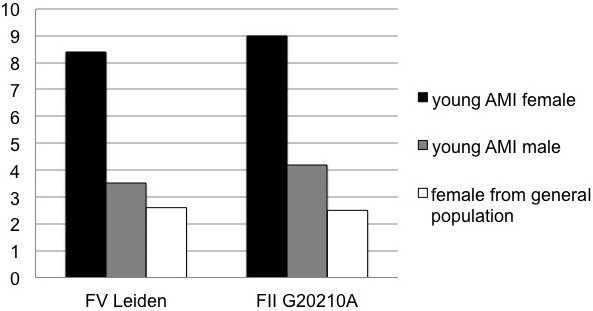
Allelic frequency (%) of the FV Leiden and FII G20210A in young AMI females, males and in female from the general population.

Table 2 reports the allele frequencies of the four variants (FV Leiden, FII G20210A, MTHFR C677T and -455G>A of beta fibrinogen) obtained in male and female with young AMI. As shown in Figure 1 the allelic frequency of the FV Leiden was significantly higher (p<0.001) in young AMI females (8.4%) than in young AMI males (3.5%) and in the general population (2.4%). Similarly, the allelic frequency of the G20210A variant was significantly higher (p<0.001) in young AMI females (9.0%) than in young AMI males (4.2%) and in the general population (2.5%, Figure 1). Then, FV Leiden had a odds ratio for young AMI in females of 3.67 (C.I.: 2.45-5.49), while FII G20210A had a odds ratio of 3.84 (C.I.: 2.59-5.70). As shown in the same Table, the allelic frequency of the two other prothrombotic variants (i.e., the MTHFR C677T and the -455 A>G beta fibrinogen variant) did not differ significantly in females with young AMI compared with young AMI males and with the general population.

**Table 2:** Number and allelic frequency (%) of prothrombotic gene variants in young AMI female, male and general population

**Group A**	**FV Leiden**	**FII G20210A**	**MTHFR C677T**	**Beta Fibrinogen -455 G>A**
Female	61/724 (8.4)*	65/724 (9.0)**	280/720 (39)	96/714 (13.4)
Male	41/1186 (3.5)	50/1186 (4.2)	505/1186 (42.6)	178/1098 (16.2)
General population	42/1718 (2.4)	43/1718 (2.5)	760/1818 (41.8)	186/1308 (14.2)

*Significantly different (p<0.001) versus the general population (O.R.: 3.67; C.I.: 2.45-5.49).

** Significantly different (p<0.001) versus the general population (O.R. 3.84; C.I.: 2.59-5.70).

### AMI patients

Also, we screened AMI patients for the four prothrombotic gene variants. Table 3 reports the data of allele and genotype frequency obtained in male and female AMI patients. In male AMI patients, only the allelic frequency of the MTHFR C677T variant (63.7%) was significantly different (p<0.001) versus AMI females (47.2%) and versus the general population (41.8%); the odds ratio was 2.45 (C.I.: 2.07-2.89). The allelic frequency of the three other variants, i.e., FV Leiden, FII G20210A and -455G>A variant of beta fibrinogen did not differ significantly between AMI patients and the general population. We next compared the frequency of the homozygous variant genotypes of MTHFR (C677T) and of beta fibrinogen -455G>A in male AMI patients versus AMI females and versus the general population (Table 3B); the frequency of the homozygous MTHFR C677T genotype was significantly higher (p<0.001) in male AMI patients (39.8%), than in AMI females (26.2%) and versus the general population (17.8%), the odds ratio was 3.05 (C.I.: 2.36-3.93).

**Table 3:** Allelic frequency (A) and frequency of homozygous variant patients (B) for prothrombotic gene variants in AMI female, male and general population

**Group A**	**FV Leiden**	**FII G20210A**	**MTHFR C677T**	**Beta Fibrinogen -455G>A**
Female	17/490 (3.5)	16/472 (3.4)	220/466 (47.2)	93/470 (19.8)
Male	23/906 (2.5)	25/878 (2.8)	561/880 (63.7)*	141/890 (15.8)
General population B	42/1718 (2.4)	43/1718 (2.5)	760/1818 (41.8)	186/1308 (14.2)
Female	0	0	61/233 (26.2)	17/235 (7.2)
Male	0	0	175/440 (39.8)**	27/445 (6.1)
General population	0	0	162/909 (17.8)	18/654 (2.75)

*Significantly different (p<0.001) versus the general population (O.R.: 2.45; C.I.: 2.07-2.89).

** Significantly different (p<0.001) versus the general population (O.R.: 3.05; C.I.: 2.36-3.93).

## Discussion

To our knowledge, this is the first study investigating the possible involvement of the prothrombotic gene variants as risk factors of AMI in different gender and age subjects. Our data indicate that the FV Leiden and FII G20201A variants in females represent a risk factors for young AMI, namely, patients who developed the first episode of AMI before reach the age of 45 years. The FV Leiden is associated to a resistance to the inhibitory effect of protein C that enhances a procoagulant activity, while the FII G20210A variant causes higher levels and more pronounced procoagulant activity of FII, both of which may predispose to ischemic diseases [5]. We observed a higher frequency of such variants only in young AMI females and not in young AMI males or in AMI females, confirming that young AMI females are a well-defined group of subjects with peculiar risk factors [1-3]. On the other hand, risk factors for young AMI are different from those observed in AMI, e.g., the atherosclerosis burden is less relevant in young AMI, particularly in women [4]. While, genetic factors seem to be more relevant [4,17,18]. Our data are in accordance with the results of a large study by Mannucci et al. [9] in which there was a higher (albeit not significantly different) allelic frequency of the FII G20210A variant in young AMI than in controls; however the authors did not analyze the data in terms of gender, and the group they studied contained more men than women (1680 men and 210 female). In the same study, the FV Leiden mutation was significantly more frequent in young AMI patients.

Furthermore, our data indicate that the C677T MTHFR variant confers a higher risk for AMI to males. Such variant is a known risk factor for coronary heart disease [19], but our study revealed that only male homozygous subjects for this variant have a higher odds ratio for AMI. The MTHFR C677T variant causes higher homocysteine and lower folate levels in serum, and this is particularly true in homozygous subjects. In this context, it is noteworthy that the mechanism by which high levels of circulating homocysteine or low levels of folate may contribute to AMI pathogenesis it is still obscure [20]. Interestingly, we observed a higher frequency of MTHFR C677T only in AMI males and not in young AMI patients. It is conceivable that this variant acts as a risk factor in older but not in young subjects because the latter have a better folate intake [21]. The other three prothrombotic variants we investigated (FV Leiden, FII G20201A and -455 G>A in the gene encoding the beta-fibrinogen) do not seem to contribute to AMI risk. These data are in partial discordance with previous studies that implicated these gene variants in AMI, but in most cases the sample size and the differences between patients and controls were small. Another study, in agreement with ours, excluded a major role of these variants as risk factors for AMI [17] and a large meta-analysis concluded that prothrombotic gene variants are only moderately associated with the risk of coronary diseases [9].

## Conclusion

AMI is a multifactorial disease due to the combination of many genetic and environmental risk factors; this is the first study reporting on a significant association between some prothrombotic gene variants and the occurrence of young AMI in females, confirming the peculiarity of such phenotype. These data (once confirmed in other populations) may contribute to a better stratification of the genetic AMI risk in different age and gender subjects and may have implications for the use of therapies to reverse the prothrombotic phenotype in high risk patients.

## Competing interests

All authors declare that they have no competing interests.

## Authors' contributions

All authors contributed equally to this work. RT administered the experiments and wrote the manuscript; CB, AC, RDF, SQ designed and performed experiments; DN, ABC and FZ recorded clinical data from all subjects. PDM selected the population; MC, GC, LD designed experiments, analysed data and edited the paper. All authors discussed the results and implications and commented on the manuscript at all stages. All authors have read and approved the contents of the paper.

## References

[B1] Choudhury L Marsh JD Myocardial infarction in young patients Am J Med 1999 107 254 261 10.1016/S0002-9343(99)00218-1 10492319

[B2] Doughty M Mehta R Bruckman D Das S Karavite D Tsai T Eagle K Acute myocardial infarction in the young-The University of Michigan experience Am Heart J 2002 143 56 62 10.1067/mhj.2002.120300 11773912

[B3] Fournier JA Cabezón S Cayuela A Ballesteros SM Cortacero JA Díaz De La Llera LS Long-term prognosis of patients having acute myocardial infarction when </=40 years of age Am J Cardiol 2004 94 989 992 10.1016/j.amjcard.2004.06.051 15476609

[B4] Marenberg ME Risch N Berkman LF Floderus B De Faire U Genetic susceptibility to death from coronary heart disease in a study of twuins N Engl J Med 1994 330 1041 1046 10.1056/NEJM199404143301503 8127331

[B5] Bafunno V Margaglione M Genetic basis of thrombosis Clin Chem Lab Med 2010 48 S41 S51 21034258 10.1515/CCLM.2010.361

[B6] Kyrle PA Rosendaal FR Eichinger S Risk assessment for recurrent venous thrombosis Lancet 2010 376 2032 2039 10.1016/S0140-6736(10)60962-2 21131039

[B7] Di Micco P Di Fiore R Niglio A Quaranta S Angiolillo A Cardillo G Castaldo G Different outcome of six homozygotes for prothrombin A20210A gene variant J Transl Med 2008 15 36 18627609 10.1186/1479-5876-6-36 PMC2483266

[B8] Kathiresan S Yang Q Larson MG Camargo AL Tofler GH Hirschhorn JN Gabriel SB O'Donnell CJ Common genetic variation in five thrombosis genes and relations to plasma hemostatic protein level and cardiovascular disease risk Arterioscler Thromb Vasc Biol 2006 26 1405 1412 10.1161/01.ATV.0000222011.13026.25 16614319

[B9] Zheng Y Liu EHC Higgins JPT Keavney BD Lowe GDO Collins R Danesh J Seven haemostatic gene polymorphisms in coronary disease: meta-analysis of 66155 cases and 91307 controls Lancet 2006 367 651 658 10.1016/S0140-6736(06)68263-9 16503463

[B10] Mannucci PM Asselta R Duga S Guella I Spreafico M Lotta L Merlini PA Peyvandi F Kathiresan S Ardissino D The association of factor V Leiden with myocardial infarction is replicated in 1880 patients with premature disease J Thromb Haemost 2010 8 2116 2121 10.1111/j.1538-7836.2010.03982.x 20626623

[B11] Atherosclerosis, Thrombosis and Vascular Biology Italian Study Group No evidence of association between prothrombotic gene polymorphisms and the development of acute myocardial infarction at a young age Circulation 2003 107 1117 1122 10.1161/01.CIR.0000051465.94572.D0 12615788

[B12] Yamada Y Ichihara S Nishida T Molecular genetics of myocardial infarction Genomic Med 2008 2 7 22 10.1007/s11568-008-9025-x 18704761 PMC2518661

[B13] Ioannidis JPA Trikalinos TA Nizani EE Contopoulos-Ioannidis DG Genetic associations in large versus small studies: an empirical assessment Lancet 2003 361 567 571 10.1016/S0140-6736(03)12516-0 12598142

[B14] Thygesen K Alpert JS White HD On behalf of the Joint ESC/ACCF/AHA/WHF Task Force for the Redefinition of Myocardial Infarction. Universal Definition of Myocardial Infarction Circulation 2007 116 2634 2653 10.1161/CIRCULATIONAHA.107.187397 17951284

[B15] Scudiero O Nardone G Omodei D Tatangelo F Vitale DF Salvatore F Castaldo G A mannose-binding lectin defective haplotype is a risk factor for gastric cancer Clin Chem 2006 52 1625 1627 10.1373/clinchem.2006.071696 16873315

[B16] Tomaiuolo R Ruocco A Salapete C Carru C Baggio G Franceschi C Zinellu A Vaupel J Bellia C Lo Sasso B Ciaccio M Castaldo G Deiana L Activity of mannose-binding lectin in centenarians Aging Cell 2012 11 394 400 10.1111/j.1474-9726.2012.00793.x 22239660 PMC3935210

[B17] Bellia C Tomaiuolo R Caruso A Sasso BL Zarrilli F Carru C Deiana M Zinellu A Pinna S Castaldo G Deiana L Ciaccio M Fetuin-A serum levels are not correlated to kidney function in long-lived subjects Clin Biochem 2012 45 637 640 10.1016/j.clinbiochem.2012.02.024 22425942

[B18] Boekholdt SM Bijsterveld NR Moons AHM Levi M Büller HR Peters RJG Genetic variation in coagulation and fibrinolytic proteins and their relation with acute myocardial infarction Circulation 2001 104 3063 3068 10.1161/hc5001.100793 11748101

[B19] Morita H Taguchi J Kurihara H Kitaoka M Kaneda H Kurihara Y Maemura K Shindo T Minamino T Ohno M Yamaoki K Ogasawara K Aizawa T Suzuki S Yazaki Y Genetic polymorphism of 5,10-methylenetetrahydrofolate reductase (MTHFR) as a risk factor for coronary artery disease Circulation 1997 95 2032 2036 10.1161/01.CIR.95.8.2032 9133512

[B20] Meleady R Ueland PM Blom H Whitehead AS Refsum H Daly LE Vollset SE Donohue C Giesendorf B Graham IM Ulvik A Zhang Y Bjorke Monsen AL EC Concerted Action Project: Homocysteine and Vascular Disease Thermolabile methylenetetrahydrofolate reductase, homocysteine, and cardiovascular disease risk: the European Concerted Action Project Am J Clin Nutr 2003 77 63 70 12499324 10.1093/ajcn/77.1.63

[B21] Klerk M Verhoef P Clarke R Blom HJ Kok FJ Schouten EG MTHFR Studies Collaboration Group MTHFR 677C>T polymorphism and risk of coronary heart disease: a meta-analysis JAMA 2002 288 2023 2031 10.1001/jama.288.16.2023 12387655

